# Enhancing surgical planning for abdominal tumors in children through advanced 3D visualization techniques: a systematic review of future prospects

**DOI:** 10.3389/fped.2024.1386280

**Published:** 2024-05-07

**Authors:** Pauline Lopez, Alexis Belgacem, Sabine Sarnacki, Alexis Arnaud, Jenna Houari, Christophe Piguet, Maxime Baudouin, Laurent Fourcade, Thomas Lauvray, Quentin Ballouhey

**Affiliations:** ^1^ Service de Chirurgie Viscérale Pédiatrique, Hôpital des Enfants, Limoges Cedex, France; ^2^ Service de Chirurgie Pédiatrique Viscérale, Urologique et Transplantation, Hôpital Necker-Enfants Malades, Assistance Publique-Hôpitaux de Paris, Université Paris Cité, Paris, France; ^3^ Service de Chirurgie Pédiatrique, CHU Rennes, Institut NuMeCan, INRAe, INSERM, Univ Rennes, Rennes, France; ^4^ Service d’Oncologie Pédiatrique, Hôpital des Enfants, Limoges Cedex, France; ^5^ Service de Radiologie Pédiatrique, Hôpital des Enfants, Limoges Cedex, France

**Keywords:** pediatric oncology, three-dimensional printing, virtual reality, augmented reality, minimally invasive surgery

## Abstract

**Introduction:**

Preoperative three-dimensional (3D) reconstruction using sectional imaging is increasingly used in challenging pediatric cases to aid in surgical planning. Many case series have described various teams' experiences, discussing feasibility and realism, while emphasizing the technological potential for children. Nonetheless, general knowledge on this topic remains limited compared to the broader research landscape. The aim of this review was to explore the current devices and new opportunities provided by preoperative Computed Tomography (CT) scans or Magnetic Resonance Imaging (MRI).

**Methods:**

A systematic review was conducted to screen pediatric cases of abdominal and pelvic tumors with preoperative 3D reconstruction published between 2000 and 2023.

**Discussion:**

Surgical planning was facilitated through virtual reconstruction or 3D printing. Virtual reconstruction of complex tumors enables precise delineation of solid masses, formulation of dissection plans, and suggests dedicated vessel ligation, optimizing tissue preservation. Vascular mapping is particularly relevant for liver surgery, large neuroblastoma with imaging-defined risk factors (IDRFs), and tumors encasing major vessels, such as complex median retroperitoneal malignant masses. 3D printing can facilitate specific tissue preservation, now accessible with minimally invasive procedures like partial nephrectomy. The latest advancements enable neural plexus reconstruction to guide surgical nerve sparing, for example, hypogastric nerve modelling, typically adjacent to large pelvic tumors. New insights will soon incorporate nerve plexus images into anatomical segmentation reconstructions, facilitated by non-irradiating imaging modalities like MRI.

**Conclusion:**

Although not yet published in pediatric surgical procedures, the next anticipated advancement is augmented reality, enhancing real-time intraoperative guidance: the surgeon will use a robotic console overlaying functional and anatomical data onto a magnified surgical field, enhancing robotic precision in confined spaces.

## Introduction

Pediatric oncologic surgery is often considered the pinnacle for pediatric surgeons, given its critical impact on clinical outcomes, particularly patient survival and quality of life (1, 2). Advances in minimally invasive surgery have led to promising improvements in safety and precision, thanks to emerging techniques such as robotic platforms (3) and fluorescence-guided surgery (4). Surgical planning remains crucial to assess the feasibility of adhering to oncologic principles, such as achieving en-bloc macroscopically complete resection or preserving neighboring organs, while also avoiding injury to adjacent vital structures.

3D technology proves to be a valuable tool, as evidenced in cases series, providing additional information and reducing surgical complications compared to relying solely on 2D information (5, 6). Although 3D modelling and printing are not yet standardized in clinical practice, their popularity is on the rise, given their demonstrated positive impact on clinical outcomes in the adult population (7). For pediatric cases, particularly complex ones like neuroblastoma with IDRFs, they are deemed indispensable, because they offer intraoperative tumor anatomy reconstruction and facilitate guidance during procedures (8).

The delineation of tumors and adjacent organs enables a comprehensive understanding of the region of interest through a 3D physical or virtual model. This enhances comprehension of the underlying structures, facilitates simulation of the resection line, and aids in procedure planning.

The initial phase of 3D surgical planning involves the acquisition of images by radiologists. Computed Tomography (CT scan) and Magnetic Resonance Imaging (MRI) images are converted to Digital Imaging and Communications in Medicine (DICOM) format. Specific post-processing medical imaging techniques, such as Multiplanar Reconstruction, Volume Rendering (VolR), and Cinematic Rendering (CR), are then utilized to automatically convert the standardized 2D images into 3D images for projection on a screen (9).

Following image conversion, DICOM data segmentation is performed through collaboration among radiologists, surgeons and bioengineers. This segmentation process involves the use of specific software to reconstruct the DICOM data into 3D models, which can be further utilized for techniques like 3D printing, virtual reality (VR), and augmented reality (AR). Since 2004 (10), numerous surgical practitioners have documented their experiences in surgical planning for abdominal pediatric tumors, utilizing 3D modelling, 3D printing, and VR technologies.

The aim of this review was to describe the various 3D surgical planning techniques and to highlight their respective benefits, limitations, and potential applications.

## Material and methods

### Search strategy

Two authors (PL and QB) independently conducted a literature search of the MEDLINE/PubMed/Cochrane online databases using the following terms: Term 1—“pediatric oncology” OR “Wilms tumor” OR “pediatric abdominal tumor” OR “pediatric abdominal tumor” OR “Neuroblastoma” OR “retroperitoneal tumor” OR “Hepatoblastoma” OR “Germ cell tumor” AND Term 2—“3D reconstruction” OR “3D modelling” OR “3D printing” OR “augmented reality” OR “virtual reality” or “mixed reality”. All relevant studies published between 2000 and 2023 were retrieved, and duplicates were removed upon identification. Filters were applied to limit results to the English language, human research, and publications from the year 2000 onwards.

### Publication selection

The inclusion criteria for the search were as follows: (i) mean participant age under 18 years; (ii) patient population undergoing a surgical procedure; (iii) 3D reconstruction focusing on preoperative tumor visualization; (iv) tumor located in the abdominal or pelvic region.

Exclusion criteria included review articles and conference abstracts, studies related to thoracic, limb, or brain tumors, as well as those concerning protheses and tissue constructs. Duplicates were removed upon identification. Publications written in languages other than English or without full paper available were excluded based on the abstract.

### Data extraction

Two reviewers (PL, QB) conducted independent searches and compared results after assessing all identified abstracts for compliance with the review criteria. In cases where agreement could not be reached, a third independent reviewer (AB) was consulted. Reasons for exclusion were documented.

The following data were extracted from the eligible studies: sample size, mean age, country of origin of the study population, study design, type of imaging performed, tumor and tumor location, type of surgery performed, and complications.

## Results

**Figure 1:**
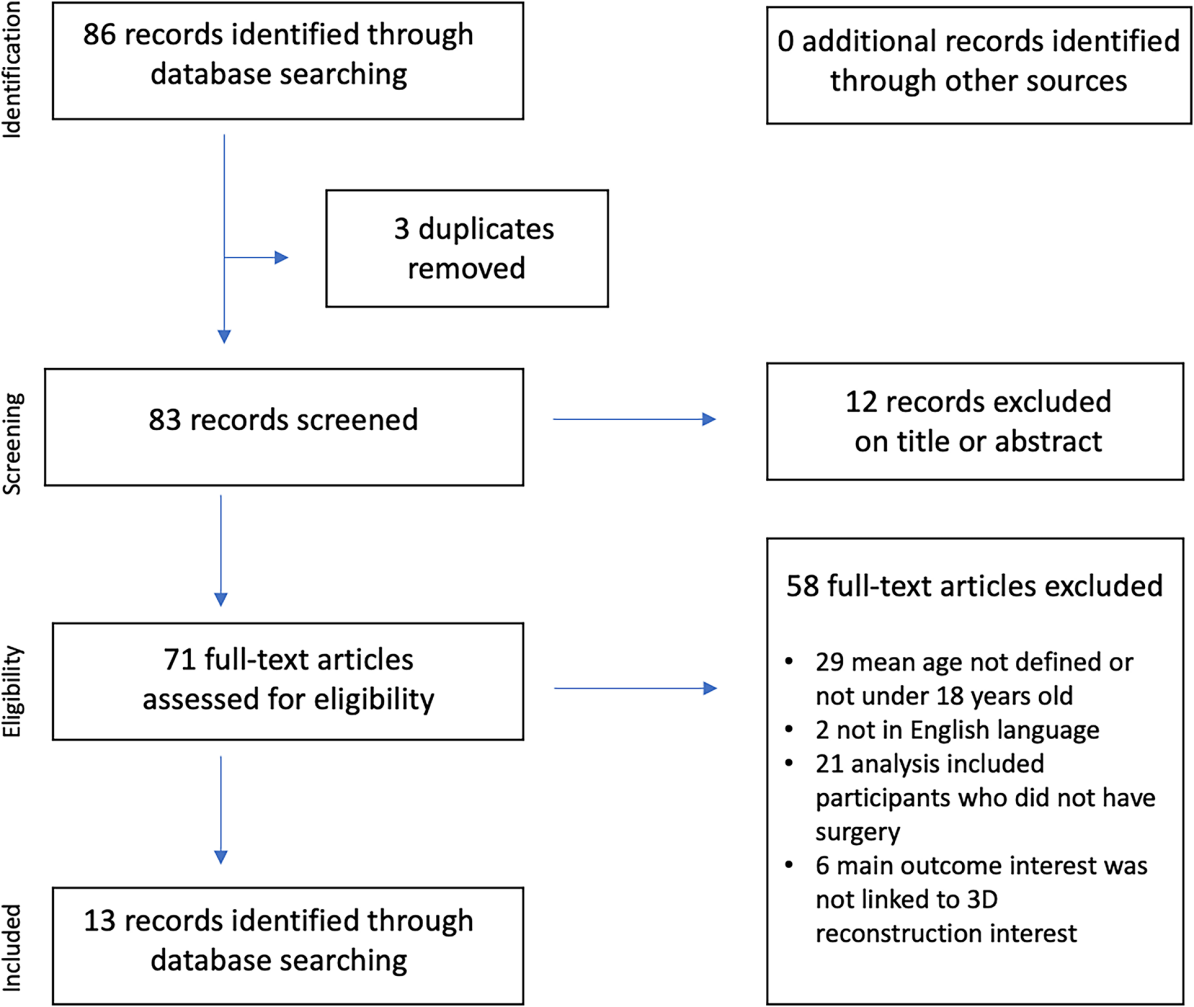
PRISMA flow diagram of the literature review.

**Figure 2:**
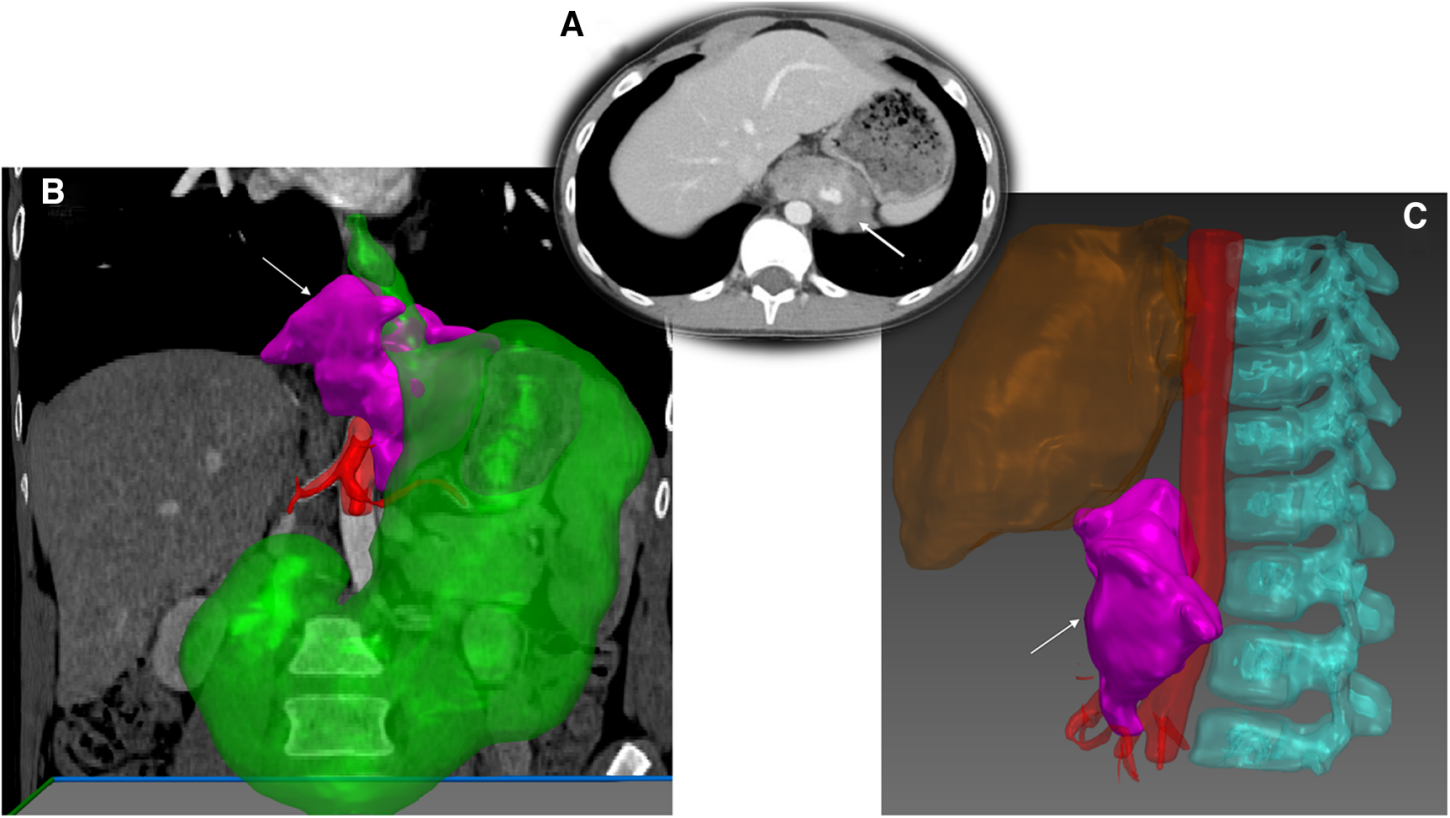
Abdominal inflammatory myofibroblastic tumor and 3D modelling. (**A**) Computed tomography acquisition of a retroperitoneal mass in a 12-year-old boy. (**B**) Segmentation of the tumor (arrow) using Medical Imaging Interaction Toolkit (MITK°-free open source software-German Cancer Research-Germany) showing close contact with abdominal aorta (**C**).

**Figure 3:**
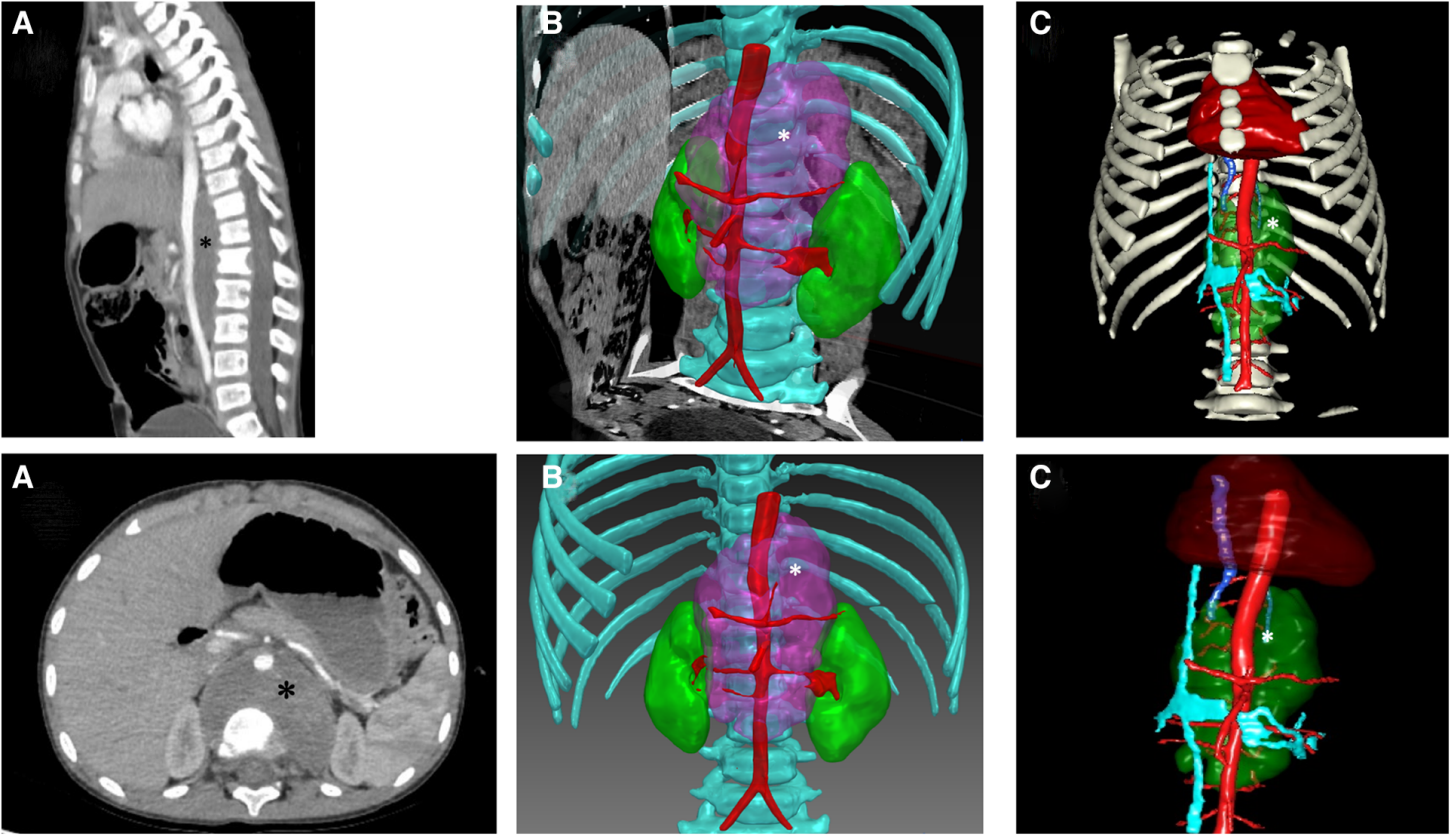
Abdominal neuroblastoma and 3D modelling. (**A**) Computed tomography acquisition of an abdominal median neuroblastoma (*) in a 4-year-old boy. (**B**) Segmentation of the tumor (*) using Medical Imaging Interaction Toolkit (MITK°-free open source software-German Cancer Research-Germany) located between vertebra and aorta. (**C**) Segmentation of the neuroblastoma (*) and surrounding structures by Visible Patient° software (Visible Patient°-Strasbourg-France).

**Figure 4:**
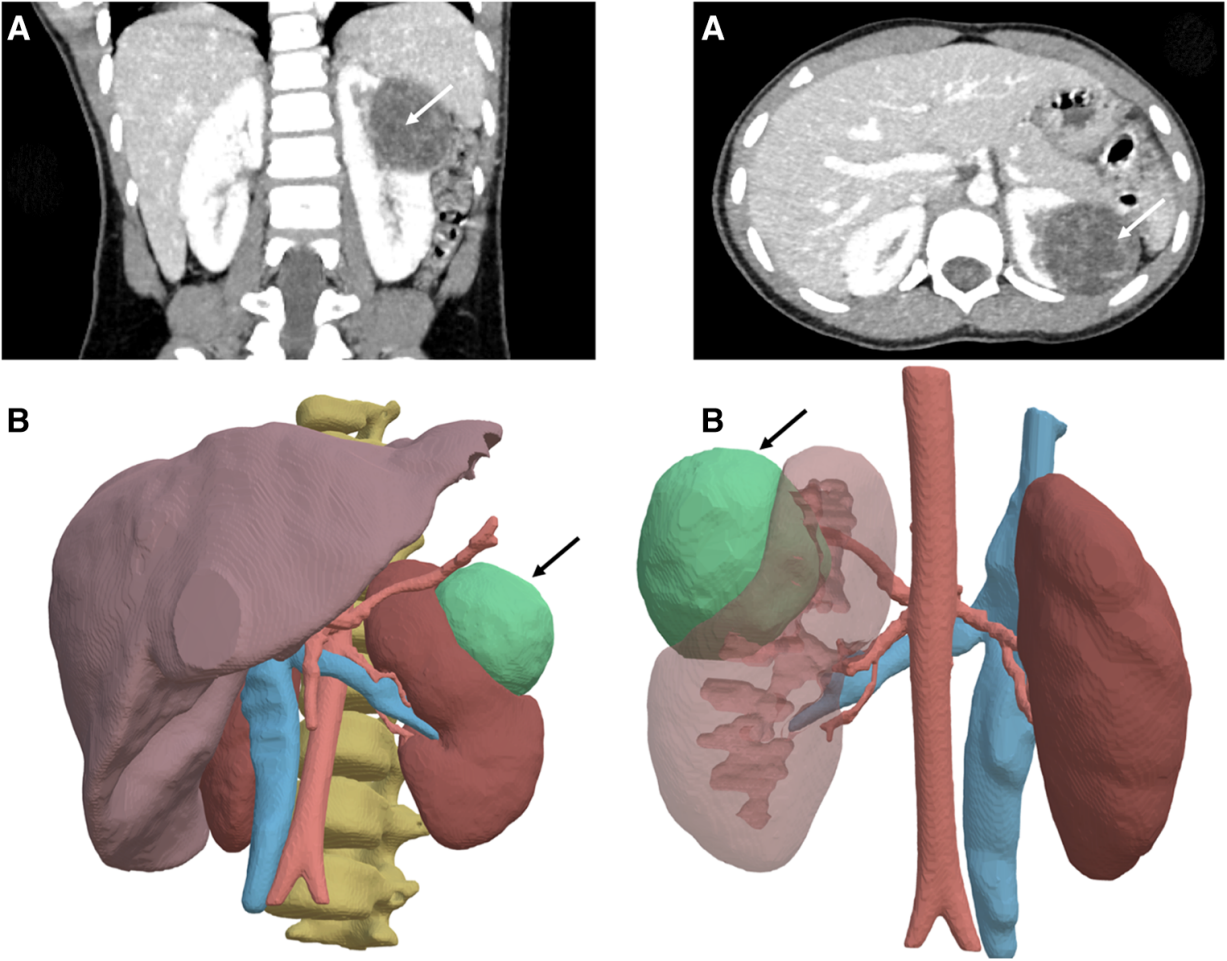
Left renal tumor and 3D modelling. (**A**) Computed tomography acquisition of a cystic nephroma in a 5-year-old boy with DICER1 syndrome. (**B**) Segmentation of the tumor (arrow) using semi-automatic methods in the IMAG2 laboratory-Paris-France.

Our search across the various databases yielded 86 articles, with 3 duplicates. Of these, 71 were assessed for eligibility, but only 13 met our criteria (Figure 1). No cases involving virtual or mixed reality were found in the literature. We did not uncover any instances of surgical resection using robotic laparoscopic assistance or cases of augmented reality applied to abdominal tumor resection in children. There were no documented cases of fetal tumor 3D exploration using MRI. Among the 13 articles identified, 4 pertained to virtual reconstruction (5, 10–12), and 10 articles focused on 3D printing, potentially in conjunction with virtual reconstruction (13–22). In the first group of studies, the surgical team did not have access to physical models of the tumor or anatomical structures. In the second group, no standardized printing systems were recommended. These pediatric studies are detailed in Table 1, comprising retrospective cases series with over 20 patients, totaling 87 cases with a mean age of 40.5 (±27.2) months. Three patients from our centers were included in this series to illustrate the discussion (Figures 2–4), bringing the total number of patients to 90. Four main groups of abdominal tumors were identified: neuroblastoma, Wilms Tumor, liver tumors, and others such as germ cell tumors and myofibroblastic inflammatory tumors.

**Table 1:** 3D support for abdominal tumors in children.

Indications	Studies	Years	Number of patients	Age (months)	Imaging	3D application	Surgical technique	Complication
				Mean				(%)
Neuroblastoma	Günther et al. (10)	2004	8	22	MRI	VR	Laparotomy	No
	Günther et al. (5)	2008	6	27	MRI	VR	Laparotomy	No
	Souzaki et al. (13)	2015	3	14	CT	PM	LS	No
	Krauel et al. (14)	2016	2	48	CT and MRI	PM	Laparotomy	
	Sanchez-Sanchez et al. (15)	2018	2	36	MRI	PM	Laparotomy	Renal artery thrombosis
	Irtan et al. (11)	2020	7	68	CT	VR	Laparotomy and LS	Extensive blood loss
	Tejo et al. (16)	2021	1	NS	CT	PM	Laparotomy	NS
	Tejo-Otero et al. (17)	2021	1	36	CT	PM	Laparotomy	NS
	Current study-Figure 2	2024	1	42	CT	VR	Laparotomy	NS
Total and median (IQR)			29	36 (range 13–66)				7%
Wilms tumors	Günther et al. (10)	2004	6	34	MRI	VR	Laparotomy	Opening of the tumor
	Günther et al. (5)	2008	5	30	MRI	VR	Laparotomy	No
	Giron-Vallejo et al. (18)	2018	1	NS	MRI	PM		No
	Sanchez-Sanchez et al. (15)	2018	1	7	MRI	PM	Laparotomy	No
	Wellens et al. (19)	2019	10	43	CT and MRI	PM	LS	No
	Irtan et al. (11)	2020	7	68	CT	VR	Laparotomy and LS	No
	Current study-Figure 3	2024	1	64	CT	VR	Robotic LS	No
Total and Median (IQR)			31	36 (range 18–68)				3.2%
	Souzaki et al. (20)	2015	1	36	CT	PM	Laparotomy	NS
Liver tumors	Yang et al. (21)	2018	1	NS	CT	PM	Laparotomy	NS
	Su et al. (12)	2016	16	11	CT	VR	Laparotomy	No
	Tejo-Otero et al. (22)	2020	1	NS	CT	PM	Laparotomy	NS
	Irtan et al. (11)	2020	4	68	CT	VR	Laparotomy and LS	No
Total and Median (IQR)			23	10 (range 4–34)				0%
Others	Irtan et al. (11)	2020	6	68	CT	VR	Laparotomy and LS	Incomplete resection
	Current study-Figure 1	2024	1	148	CT	VR	Laparotomy	NS
Total and Median (IQR)	14 studies		90	36 (range 12–148)		4 exclusive VR		4.4%

Summary of imaging modalities and 3D applications used in various studies.

NS, not specified; LS, laparoscopy; VR, virtual reconstruction; PM, printed model.

Age is expressed in median (range IQR1, IQR3).

All authors reached the consensus that the utilization of both virtual reconstruction technology and physical model printing proved to be beneficial tools for enhancing surgical planning and improving patient outcomes in cases of complex tumors. However, to date, there is a lack of evidence-based medicine arguments to substantiate these conclusions or to support the funding of such technologies and the selection of candidate patients.

## Discussion

### 3D modelling

3D virtual reconstruction offers numerous applications, including monitoring tumor volume during chemotherapy (11), adjusting the target irradiation volume (23), and providing precise preoperative visualization of the tumor for the surgical team. This process can be performed “*in situ*” by radiologists, resulting in the projection of 3D images on the screen (9). The primary technology used for this propose is VolR, available with conventional software (24). It serves as an effective tool, as demonstrated in pelvic region analysis for adults, where it provides additional information beyond standard MRI (25). However, the limitation of this pseudo-3D technology lies in the lack of accurate tissue differentiation, such as distinguishing between normal tissue and tumors. It often necessitates cross-referencing with CT or MRI images (Figure 2).

A more comprehensive process is offered by engineering companies providing remote modeling services like “Visible Patient°” (11). Technicians delineate various anatomical structures and return files with reconstructed tumors and surrounding anatomical features. This service offers a precise vascularization map, providing valuable information about potential encasement of large vessels encasement that is crucial for surgical planning and the mental preparation of the surgical team. Figure 3 illustrates the segmentation of a median neuroblastoma with IDRFs.

Vessels can be delineated within the application, allowing identification of the feeding artery corresponding to the tumor. Simulations of vessel clamping with tissue ischemia can be performed. This technology has been reported to prevent cold intraoperative ischemia in adult patients (26) and can also estimate the resultant organ volume for hepatectomy (27). More recently, the IMAG2 laboratory (Imagine Institute, Necker Enfants Malades Hospital, APHP and Université Paris Cité-France) has developed semi-automatic segmentation methods, combining artificial intelligence methods (knowledge representation, spatial reasoning and deep learning) making it possible to obtain automatic 3D modeling of bones, bladder, colon, vessels, pelvic muscles (obturator, levator ani, piriformis), genital tract in adolescents (ovaries, vagina, uterus), and nerves in a few clicks. This methodology also applied to renal tumor CT scan images making it possible to quickly model 3D structures which cannot benefit from automatic learning due to their excessive inter-individual variations, like tumors (Figure 4) (24, 28, 29). All the aforementioned points contribute to the preoperative liver 3D evaluation being covered by universal health care insurance in Japan since 2012 (30).

Some masses are embryonal abdominal tumors and can be detected prenatally. Fetal MRI is more precise than sonography, and while T2 sequences are well-developed for placental research, they are not superficially outlined for fetal applications. Consequently, even though MRI is the most efficient tool for describing prenatal malformations and providing accurate anatomical delineation of cystic lesions (31), 3D fetal tumor reconstruction is not commonly performed.

#### 3D printing

Physical model printing is an additional option after 3D reconstruction. Initially developed for bones tissues and prothesis implantation, it is now common in adult surgeries such as orthopedics (32). The first surgical 3D printing pediatric case involved a heart model before transplantation (33) and has since expanded to utilize multiple materials. In case of tissue lesions, segmentation allows for precise delineation from other soft tissues like vascular structures. It has been reported as useful in enhancing partial nephrectomy in adults (34), with volumetric precision sufficient for distinguishing between nephron sparing surgery or total nephrectomy (15, 19). Real-scale models can facilitate familial preoperative counseling and, from an educational perspective, can be manipulated by the entire surgical team to improve understanding of abdominal anatomy (35). Most importantly, it can be used for realistic surgical laparoscopic simulation (13) through the assembly of several semi- transparent materials.

The main limitations include its difficulty in use during the intraoperative process without the distracting intervention of a non-sterile person, the lack of reusability, its availability including printing duration (ranging from 4 h to 5 days), and its cost (ranging from $30 to $900) (36). Although a recent study proposed a recommendation guide for sterilization methods of 3D-printed materials (37), its advantages compared to 3D virtual reconstruction are expected to be proven with large series.

#### Augmented reality

**Figure 5:**
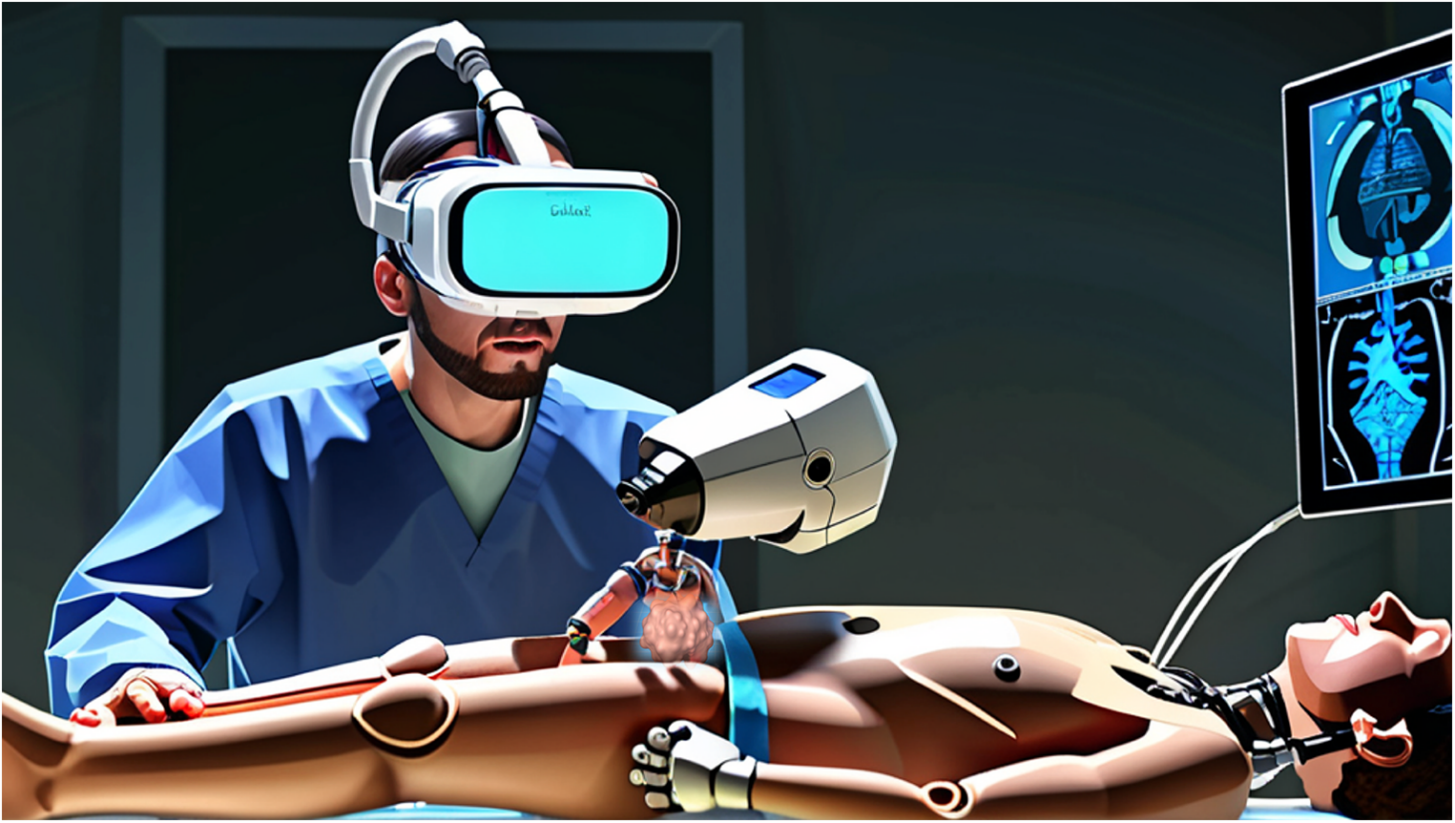
Augmented reality. Illustration of a potential operating room equipped with augmented reality for a pediatric surgery case in the future.

AR involves integrating information about the surgical field into the surgeon's mind to assist with the procedure, while allowing the surgeon to maintain direct contact with the environment. On the other hand, VR remains a futuristic concept for open surgical processes, as it requires full immersion with a headset (Figure 5). To the best of our knowledge, this approach has not yet been published for pediatric patients, but it could have potential applications in open surgery. AR allows for overlaying digital information onto the physical real world, making it useful for surgical navigation (38) and education.

Before AR was generated by specific software, blending the surgical field with additional information was done intraoperatively by the surgeon's brain. Robotic gamma detection of neuroblastoma was reported for intraoperative resection support before the laparoscopic era (39). Some methods are now available using the laparoscopy screen. Fluorescence-guided surgery (4) using indocyanine green enhances perception of vascularization in the operative field, and a dedicated robotic interface for near-infrared fluorescence-guided surgery has been reported to be effective for partial nephrectomy and tumor excision guidance (40). Intraoperative use of an ultrasound probe has been reported for pancreatic surgery, providing both visual and ultrasound images (41) integrated into the robotic console. In a conference paper, Stafman et al. reported that intraoperative ultrasound guidance facilitates laparoscopic resection of smaller, non-visible tumors and optimizes negative margins (42). Others technologies like photodynamic therapy and near-infrared photoimmunotherapy show promising results (43). However, integrating these devices into standard laparoscopy seems less pertinent due to the lack of 3D visualization and precision of movements compared to robotic surgery. In the only case included in this review that was operated with the robot, AR was not available at the console.

**Figure 6:**
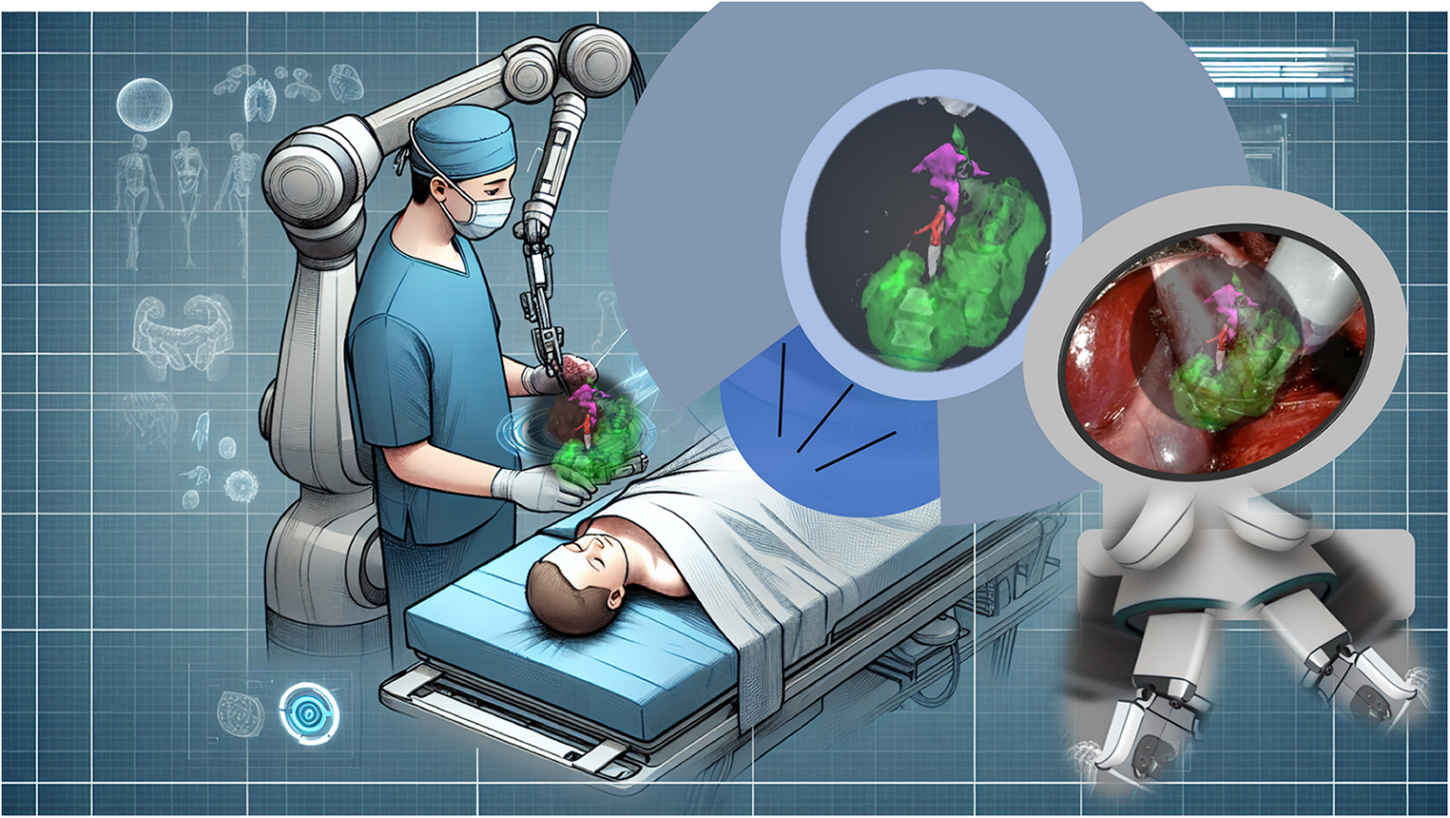
Mixed reality. Illustration depicting a potential scenario of mixed reality support for a pediatric surgery case in the future.

Mixed reality combines AR and VR to create a new operating field (Figure 6). Its integration into the robotic console would allow blending anatomical structures and simulated digital elements like nervous plexi, tumor delineation, and vascularization, synchronized with respiratory movements. The concept of cybernetic surgery was first proposed in a robotic liver segmentectomy using augmented reality (44). Machine learning has been demonstrated to be relevant to human expertise for real-time anatomy segmentation during laparoscopy (45), particularly for nervous structures (46). All these supportive devices will enhance the level of assistance for the procedure. The next step will be the integration of artificial intelligence in near-real-time surgery (47), enabling semi-autonomous robotic surgery under human supervision.

## Conclusion

While there is currently no evidence to suggest that 3D technology offers a positive impact on clinical outcomes for pediatric oncology, it is widely recognized by most pediatric surgeons as a key future tool, especially when used in conjunction with dedicated small instruments and robotic platforms. In the near future, pediatric patients are expected to benefit from systematic surgical planning, which may include 3 modeling prior to minimally invasive resection procedures under mixed reality, facilitated by artificial intelligence algorithms.
